# The level of CD147 expression correlates with cyclophilin-induced signalling and chemotaxis

**DOI:** 10.1186/1756-0500-4-396

**Published:** 2011-10-11

**Authors:** Alexander Trachtenberg, Tatiana Pushkarsky, Shannon Heine, Stephanie Constant, Beda Brichacek, Michael Bukrinsky

**Affiliations:** 1 The George Washington University, Washington, DC 20037, USA; 2 NHLBI, National Institutes of Health, Bethesda, MD 20892, USA; 3 NCI, National Institutes of Health, Bethesda, MD 20892, USA

## Abstract

**Background:**

Previous studies identified CD147 as the chemotactic receptor on inflammatory leukocytes for extracellular cyclophilins (eCyp). However, CD147 is not known to associate with signal transducing molecules, so other transmembrane proteins, such as proteoglycans, integrins, and CD98, were suggested as receptor or co-receptor for eCyp. CD147 is ubiquitously expressed on many cell types, but relationship between the level of CD147 expression and cellular responses to eCyp has never been analyzed. Given the role of eCyp in pathogenesis of many diseases, it is important to know whether cellular responses to eCyp are regulated at the level of CD147 expression.

**Results:**

Here, we manipulated CD147 expression levels on HeLa cells using RNAi and investigated the signalling and chemotactic responses to eCypA. Both Erk activation and chemotaxis correlated with the level of CD147 expression, with cells exhibiting low level expression being practically unresponsive to eCypA.

**Conclusions:**

Our results provide the first demonstration of a chemotactic response of HeLa cells to eCypA, establish a correlation between the level of CD147 expression and the magnitude of cellular responses to eCypA, and indicate that CD147 may be a limiting factor in the receptor complex determining cyclophilin-induced Erk activation and cell migration.

## Background

Leukocyte trafficking and recruitment is a critical component of inflammation-mediated pathology. The main regulators of leukocyte trafficking are chemokines, a family of chemoattracting cytokines that control cell migration and adhesion [1]. However, other factors, in particular extracellular cyclophilins (eCyp), also induce potent chemotactic responses of immune cells (reviewed in [2]). We [3-6] and others [7,8] have observed that secreted cyclophilin A (CypA) is a potent leukocyte chemoattractant *in vitro *and *in vivo*.

In studies aimed at establishing the mechanism whereby cyclophilins mediate their chemotactic activity, our group identified CD147 (also known as EMMPRIN and basigin), as the signalling receptor for eCyp [3,9]. Indeed, all human [3,6] and mouse [4,5] leukocytes examined to date require expression of CD147 for cyclophilin-dependent chemotaxis to occur. However, CD147 is not known to mediate signal transduction events, suggesting that other molecule(s) associated with CD147 may function as a signal-transducing co-receptor. Consistent with this notion, truncation of the cytoplasmic tail of CD147 does not abolish signalling response to eCypA [10]. The possibility of additional co-receptor is also supported by inability of eosinophils to respond to eCypA despite the fact that these cells express relatively high levels of CD147 [5]. Such signal-transducing activity may be exerted by known CD147-interacting proteins, such as β1 integrins [11,12], CD98 [13], and syndecan-1 [14], which may function as a multi-molecular receptor complex [13]. Whether and how the level of CD147 expression influences activity of this complex is unknown. We recently reported that activated CD4^+ ^T cells showed enhanced cyclophilin-mediated chemotaxis relative to resting T cells, that correlated with an upregulated expression of CD147 [5,6], but cell activation likely increases expression of many other proteins, including those that may be involved in eCyp-induced signalling, thus precluding reliable interpretation of this result.

The chemotactic activity of CD147 has been investigated until now only for inflammatory leukocytes. However, CD147 is an ubiquitously expressed protein present on almost all studied cell types, including cancer cells and stem cells [15,16], suggesting that eCyp-induced chemotaxis may underlie such processes as metastasis or cell development. An important question in this regard is whether such response depends on the level of CD147 expression. In this study we investigated cyclophilin-induced chemotactic activity of HeLa cells manipulated by RNAi technology to express varying levels of CD147. Our results demonstrate a direct correlation between CD147 expression and signalling and chemotactic responses to eCypA, suggesting that CD147 may be a rate-limiting component in the eCypA-responding receptor complex.

## Methods

### RNA interference and CD147 knockdown

siRNA (small interfering RNA) was derived from the nucleotide sequence of CD147 [17] corresponding to nucleotides 470-490. Forward 5'-GATCCCCcggctccgagagcaggttcttcaagagagaacctgctctcggagccgTTTTTGGAAA-3' and reverse 5'-AGCTTTTCCAAAAAcggctccgagagcaggttctctcttgaagaacctgctctcggagccgGGG-3' complementary oligonucletides containing the above sequence were designed according to protocol recommended for pSUPER.retro.puro expression system (OligoEngine, Seattle, WA) by the manufacturer. A mixture of both oligonucleotides (3 μg of each) was incubated in 50 μl of 100 mM NaCl and 50 mM HEPES pH 7.4 at 90°C for 4 min, and then at 70°C for 10 minutes. The mixture was then cooled down to 10°C over a period of 50 minutes. pSUPER.retro.puro vector was linearized by subsequent cleavage with HindIII and BglII. Annealed nucleotides were ligated into the linearized pSUPER.retro.puro using T4 ligase as recommended by the OligoEngine protocol creating pSuper3,4 construct. E. coli Stbl 2 competent cells (Invitrogen) were transformed with pSuper3,4 and ampicillin resistant colonies were isolated and amplified.

Purified pSuper3,4 plasmid as well as pSuper empty vector were used for transfection of exponentially growing HeLa-CD4 [18] cells. Puromycin resistant cells were examined for CD147 expression by flow cytometry. pSuper3,4-transformed cell population was further enriched for cells with downregulated CD147 surface expression by removal of cells with higher CD147 expression using magnetic beads with conjugated anti-CD147 antibodies (Ancell, Bayport, MN). Remaining cell population was expanded, sorted on a flow cytometer, and several cell clones with low CD147 expression were established.

### Reagents

FITC-conjugated anti-CD147 mouse monoclonal antibody was purchased from Ancell. FITC-conjugated mouse IgG1 was obtained from PharMingen (BD Biosciences, San Jose, CA). Human recombinant cyclophilin A was purchased from Calbiochem (EMD, Rockland, MA). Human SDF-1α was purchased from Peprotech (Rocky Hill, NJ). The primary anti-phospho-Erk1/2 rabbit monoclonal antibody (recombinant clone AW39R) was purchased from Millipore (Billerica, MA), rabbit polyclonal antibody to Erk1/2 was from Cell Signalling Technology (Boston, MA), and the secondary antibody, anti-rabbit IgG (Horseradish Peroxidase linked F(ab)_2 _fragment), was from GE Healthcare, UK.

### In vitro chemotaxis assay

Chemotactic activity was assessed using 48-well modified Boyden chambers (Neuro Probe Inc., Gaithersburg, MD) with the two compartments separated by an 8 μm polyvinylpyrrolidone free polycarbonate membrane (Neuro Probe Inc.). HeLa-CD4 cells [18], at 10^4 ^cells/well in RPMI-1640 culture medium supplemented with 1% BSA, Cohn Fraction V (Sigma-Aldrich, St. Louis, MO), were added to the upper compartment (40 μl volume) and medium containing recombinant human CypA (100 ng/ml) or SDF-1α (50 ng/ml) or medium alone (25 μl) was added to the lower compartment. The chambers were incubated for 50 minutes at 37°C. Then the membrane was removed, nonmigrated cells were scrapped off the upper side with PBS, and the membrane, after drying, was stained with Wright-Giemsa (Camco, Fort Lauderdale, FL) to reveal bound cells. A chemotaxis index was calculated for each test well by dividing the number of cells counted for that well by the number of cells counted in media wells.

### Flow cytometry

Cells were stained with 10 μg/ml of FITC-conjugated anti-human CD147 mAb (Ancell, Bayport, MN) or FITC-IgG1 isotype control mAb for 15 min at 4°C, washed with cold PBS and used for analysis. Flow cytometric analysis was done using a FACSCalibur instrument and CellQuest Pro software (Becton Dickinson, San Jose, CA). To analyze the staining of CD147, cells were first gated by forward and side scatter. Live cell populations were gated upon on an acquisition plot and 20,000 events were collected from this region. An acquisition histogram plot was also assessed for this region using parameter 3, the FL-1 detector, set to Log mode in order to detect fluorescence of the green fluorophore, FITC. The voltage of the parameter was adjusted to the relative fluorescence intensity of the high CD147 expressing cell line. All subsequent samples were acquired using the same settings. Isotype-stained cells were used as control; background staining was less than 1%.

### Signalling analysis

Serum-starved 5 × 10^6 ^HeLa-CD4 cells were pre-treated with CypA (100 ng/ml) or PMA (100 nM), and incubated at 37°C for various time periods. Cells were lysed in Laemmli sample buffer (BioRad, Hercules, CA), sonicated for 5-7 seconds to shear DNA and reduce sample viscosity, separated on 10-20% SDS-PAGE and subjected to Western blotting analysis using anti-p44/p42 MAPK antibody and anti-phosphorylated-p44/p42 pMAPK antibody.

### Statistical analysis

Data are presented as mean ± S.E.M. Statistical analysis was performed using Student's t-test and p < 0.05 was considered significant.

## Results and discussion

**Figure 1:**
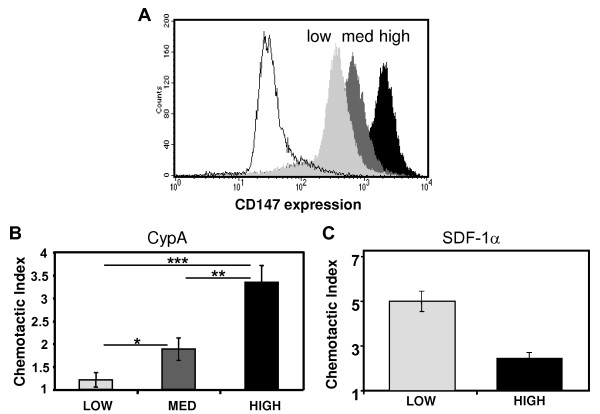
**CD147 expression and chemotactic activity**. HeLa-CD4 cells stably transfected with anti-CD147 siRNA-expressing vector or empty vector control were sorted according to the level of CD147 expression into low, medium and high (cells transfected with empty vector) expressing clones (A). Chemotactic activity of the clones in response to CypA (B) or SDF-1α (C) was measured as described in Materials and Methods. Chemotactic activity was analyzed in triplicate and results are presented as mean ± SEM, *p < 0.05; **p < 0.01; ***p < 0.001. Results are shown for one representative experiment out of two performed.

To manipulate CD147 expression levels on HeLa cells we used the RNAi knockdown approach. HeLa-CD4 (MAGI) cells were transfected with the pSuper-retro vector (OligoEngine, Seattle, WA) containing puromycin resistance gene and siRNA corresponding to the nucleotides 470-490 of CD147 mRNA. Puromycin resistant cells displayed lower concentrations of surface CD147 than parental cell line. The cells with the highest CD147 surface expression were removed using magnetic beads followed by flow cytometric cell sorting of remaining cells. Cell clones with decreased CD147 abundance were established from the remaining cell population. Cell clones with low and medium CD147 expression were selected for further analysis in comparison to original HeLa-CD4 cell clones transduced with an empty vector (high level CD147 expressors) (Figure 1A). Importantly, the level of expression changed after several passages of cells, making it necessary to verify the expression level before each experiment.

Using HeLa-CD4 cells transduced with an empty vector, we first performed a dose response analysis to identify the optimal concentration of CypA needed to induce chemotaxis. The concentration inducing maximal chemotactic response was 100 ng/ml and was used in all subsequent experiments. A good correlation between the level of CD147 expression and the chemotactic response of the cells to CypA was observed, with significant differences in chemotactic index between low, medium, and high expressing cells (Figure 1B). The observed chemotactic response was dependent on CD147 as it was completely blocked when anti-CD147 mAb was added to the cells in the upper chamber. Increased cell migration was not due to chemokinesis, as no migration was observed when CypA was added to both chambers of the Boyden chamber assembly. Importantly, the level of CD147 expression did not affect the ability of cells to migrate in response to SDF-1α (Figure 1C), thus excluding the possibility that low CD147 adversely and non-specifically affected cell mobility. In fact, cells with low CD147 expression migrated to SDF-1α better than high-CD147-expressing cells. This result may reflect some antagonism between these two receptors, suggested also by their opposing reaction to TCR activation in T lymphocytes [19]. These results are consistent with previously observed correlation between CD147 expression and CypA-induced chemotaxis of CD4+ T cells [6], however, in contrast to that previous study where activated and non-activated T cells were compared, siRNA treatment of the cells in this study is unlikely to affect expression of other than CD147 molecules, thus excluding the possibility that observed differences are due to altered expression of another signalling molecule involved in response to eCypA. Our data also indicate that a certain minimal level of CD147 expression is required for chemotactic response of cells to cyclophilin.

**Figure 2:**
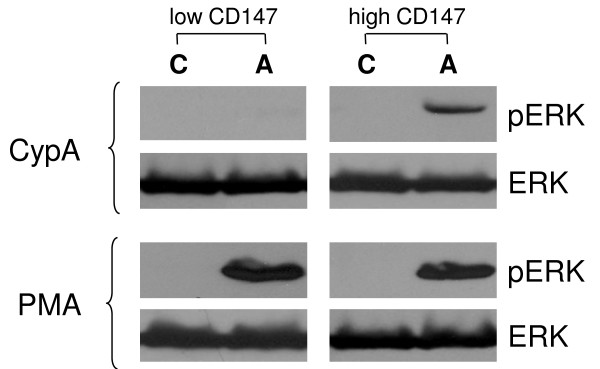
**Analysis of Erk activation**. Cells with low and high levels of CD147 expression were stimulated (A) or not (C) with CypA or PMA as described in Materials and Methods. Phosphorylated (activated) and total Erks were revealed by Western blotting. Results are presented for one representative experiment out of three performed.

Cell chemotaxis is dependent on signalling events transduced by the chemotactic receptor in response to ligand binding. Interaction of cyclophilin with CD147 induces a number of signalling events, including Ca^2+ ^mobilization and Erk 1/2 activation [3,9]. We analyzed CypA-induced Erk activation in HeLa cells expressing low and high levels of CD147. Consistent with results of the chemotaxis assay, cells with high level of CD147 expression exhibited Erk phosphorylation whereas Erk activation in cells with low level CD147 expression was minimal (Figure 2). Both high and low CD147 expressors equally responded to PMA activation, indicating that low response to eCypA was not due to an internal signalling defect. Despite the fact that the antibodies we used recognize both p42 and p44 forms of Erk, only one Erk form was detected in these experiments. This result may reflect the feature of the HeLa cell line where Erk 1 is expressed at a much lower level than Erk 2 [20]. This result establishes a correlation between the level of CD147 expression and Erk activation in response to eCypA stimulation.

Overall, results of this study provide evidence for the following two important features of the cellular responses to extracellular CypA. First, the response depends on the level of CD147 expression. Low CD147 expressing cells respond poorly to eCypA by chemotaxis or Erk activation. This dependence of response on CD147 abundance may be due to simple cumulative property of the signalling events, but may also reflect different modes of interaction between the ligand and receptor. For example, CD147, when present at low density on the cell surface, may not form homo-oligomers [21]. Although requirement of CD147 oligomerization for CypA-induced signalling has not been established, there is ample evidence for oligomerization of other chemotactic receptors [22-24]. Oligomerization of CD147 may be required to allow eCypA make contact with Proline 211 at the border of the transmembrane and ectodomain of CD147, which is the target of isomerase activity of CypA [25]. In any case, this result suggests that CD147 is the rate-limiting component of the multimeric receptor complex responding to eCypA.

Another important conclusion from this study is that the chemotaxis-inducing activity of eCypA is not limited to leukocytes but likely extends to any cell type expressing sufficient levels of CD147. This feature would be especially relevant to cancer cells, which are known to express high CD147 levels (reviewed in [26]). CypA-induced chemotaxis may be an important contributor to metastatic activity of cancer cells. Correspondingly, targeting CD147 expression level may be a valid therapeutic approach aimed at reducing metastatic activity. One of the key regulators of the level of plasma membrane expression of CD147 is Cyp60 [27,28], a member of the cyclophilin family of proteins [29]. Expression of this protein in various cells has not been characterized, but may be an important factor determining cellular responses to eCyp and a potential target for therapeutic interventions. Another potential type of effector cells responsive to extracellular CypA are stem cells [15]. Additional studies are required to correlate the level of CD147 expression on different stem cells with their chemotactic activity or other responses.

## Conclusions

Results of the current study provide the first demonstration of the chemotactic activity of HeLa cells to eCypA. They also establish a direct correlation between cellular chemotactic and signalling responses to extracellular cyclophilin A and the level of CD147 expression, suggesting that CD147 is the rate-limiting factor in the receptor complex responding to eCyp. These findings provide a strong rationale for investigating regulators of CD147 expression, which may be targeted to moderate eCyp-induced cellular responses in inflammatory diseases and metastatic activity of cancer cells.

## Competing interests

The authors declare that they have no competing interests.

## Authors' contributions

All authors read and approved the final manuscript. AT carried out cell cloning; TP performed flow cytometry and Erk activation assays; SH participated in flow cytometry analysis of CD147 expression and performed chemotaxis assays; SC contributed to the overall design of the study and to manuscript preparation; BB designed the primers for siRNA expression and contributed to cell cloning; and MB designed the study and wrote the manuscript.
